# AlN-Based Ceramic Patch Antenna-Type Wireless Passive High-Temperature Sensor

**DOI:** 10.3390/mi8100301

**Published:** 2017-10-10

**Authors:** Dan Yan, Yong Yang, Yingping Hong, Ting Liang, Zong Yao, Xiaoyong Chen, Jijun Xiong

**Affiliations:** 1 Key Laboratory of Instrumentation Science and Dynamic Measurement, Ministry of Education, North University of China, Taiyuan 030051, China; b1506004@st.nuc.edu.cn (D.Y.); hongyingping@nuc.edu.cn (Y.H.); liangting@nuc.edu.cn (T.L.); 2 Science and Technology on Electronic Test and Measurement Laboratory, North University of China, Taiyuan 030051, China; 3 Taiyuan Research Institute Co., Ltd., China Coal Technology and Engineering Group Corporation, Taiyuan 030006, China; s1606077@st.nuc.edu.cn; 4 North Automatic Control Technology Research Institute, Taiyuan 030051, China; b1406004@st.nuc.edu.cn; 5 National Demonstration Center for Experimental Chemical Engineering Comprehensive Education, North University of China, Taiyuan, 030051, China

**Keywords:** high-temperature environment, patch antenna temperature sensor, passive wireless, dielectric constant

## Abstract

An aluminum nitride (AlN) based patch antenna-type high-temperature wireless passive sensor is reported to operate as both a sensor and an antenna, which integrates in situ measurement/sensing with remote wireless communication at the same time. The sensor is small, easy to manufacture, highly sensitive and has a high operating temperature; it can be used in high-temperature, chemically corrosive and other harsh environments. The sensing mechanism of the sensor, the dielectric constant of the AlN ceramic substrate, increases with rising temperature, which reduces the resonant frequency of the sensor. Thus, the temperature can be measured by detecting changes in the sensor’s resonant frequency. High-Frequency Simulation Structure (HFSS) software is used to determine the structure and size of the sensor, which is then fabricated using thick-film technology. The substrate of the sensor is AlN ceramic due to its outstanding thermal resistance at high temperature; and its conductors (the radiation patch and the ground under the substrate) are silver-palladium alloy sintered form silver–palladium paste. A vector network analyzer reveals that the sensor’s operating range extends to 700 °C. Furthermore, its resonant frequency decreases from 2.20 GHz to 2.13 GHz with increasing temperature from room temperature (25 °C) to 700 °C, with an absolute sensitivity of 104.77 KHz/°C. Our work verifies the feasibility of measuring high temperatures using AlN-based patch antenna wireless passive temperature sensors, and provides a new material and temperature sensitive structure for high-temperature measurement in harsh environments.

## 1. Introduction

In situ real-time temperature measurement in high-temperature, high-pressure, strongly acidic or alkaline, high-radiation, and many other harsh environments is very important [1]. For example, temperature acquisition in the combustion chamber of an aircraft is conducive to improving the fuel combustion efficiency [2,3,4,5,6]; precise control of the temperature inside a nuclear reactor is conducive to maintaining the stability of the nuclear reaction; the detection of the temperature inside a steelmaking furnace is conducive to improving the quality of the steel billets.

However, it is very difficult to apply active or lead-type passive temperature sensors in these harsh environments [7]. An active sensor needs to be provided with energy by a power module, which causes problems such as the need to replace this regularly, and has a complex structure, low integration, and high fabrication costs. Furthermore, there is currently no power supply suitable for use in high-temperature environments. Lead-type passive temperature sensors require a transmission-line connection between the sensor and the signal-processing system, and the transmission line will be degraded or even destroyed with increasing temperature in a high-temperature environment [8].

Therefore, wireless passive sensing technology has been widely used and investigated [9,10,11]. Wireless passive temperature sensors adopt a non-contact measurement method and do not require a power supply device and transmission line; and they can be used in harsh environments. Surface acoustic wave (SAW), inductive–capacitive (LC), and certain microwave sensors are typical wireless passive high-temperature sensors. SAW temperature sensors work by detecting disturbances in the acoustic wave propagation characteristics caused by physical and chemical parameters, realizing detection of the measured parameters [12,13,14,15]. For example, SAW is used to measure temperature wirelessly and to characterize SiO_2_ thin films accurately [16,17,18]. However, the chemical properties of SAW substrates are unstable, limiting their application in high-temperature environments [19]. LC resonant sensors, which can be applied in harsh environments, can simultaneously measure multiple parameters, and their fabrication process is simple [20,21,22,23]. The disadvantage of such sensors is that the magnetic field is absorbed to form a vortex when they are close to a metal surface, affecting the measurement accuracy and signal transmission distance and thus limiting their practical use [24,25]. Therefore, microwave wireless passive sensors have been a focus of attention owing to their high quality, large sensing distance, lower material requirements, and other advantages. The Leonhard M. Reindl research group at the University of Freiburg achieved torque, strain, and temperature measurements by using a microwave dielectric resonator [26,27,28]. However, since the dielectric constant of the dielectric resonator is large, the emission efficiency is affected negatively. The Haiying Huang research team reported the microstrip patch antenna temperature sensor, based on Rogers materials, reaching a maximum temperature of 280 °C, beyond which the high-temperature application capacity is still needed [29,30,31]. The Ahsan Choudhuri research team presented a concept and model of a passive wireless temperature sensor based on metamaterial for harsh-environment applications, but no measurement experiments [32].The measuring range of a temperature sensor developed at Purdue University, which is based on microelectromechanical systems technology, reached only 300 °C [33]. Slotted wireless passive temperature sensors were fabricated by Wu et al. [34] and Cheng et al. [35], but they are not reliable and durable because the metal coating on side walls are difficult to process and easily fall off. A patch-type high-temperature sensor based on Al_2_O_3_ was fabricated at the University of Central Florida; it has a high operating temperature and good wireless transmission, indicating the feasibility of using patch-type high-temperature sensors [36]. However, because Al_2_O_3_ ceramic has low thermal conductivity and cannot withstand heat shock, reliable operation cannot be guaranteed. Aluminum nitride (AlN) ceramic has excellent thermal shock resistance compared to Al_2_O_3_ ceramic (demonstrated by Lucun Guo et al.) [37], due to its higher thermal conductivity (about 10 times the thermal conductivity of Al_2_O_3_ ceramic) and lower thermal expansion coefficient (about half the thermal expansion coefficient of Al_2_O_3_ ceramic) [38,39,40,41,42]. Therefore, this paper studies an AlN-based patch antenna-type wireless passive temperature sensor, and examines the high-temperature sensing applicability of the sensor for the first time. It is believed that our results provide possible uses for high-temperature sensors in ultra-high temperature and harsh environments.

## 2. Measurement Principle

**Figure 1:**
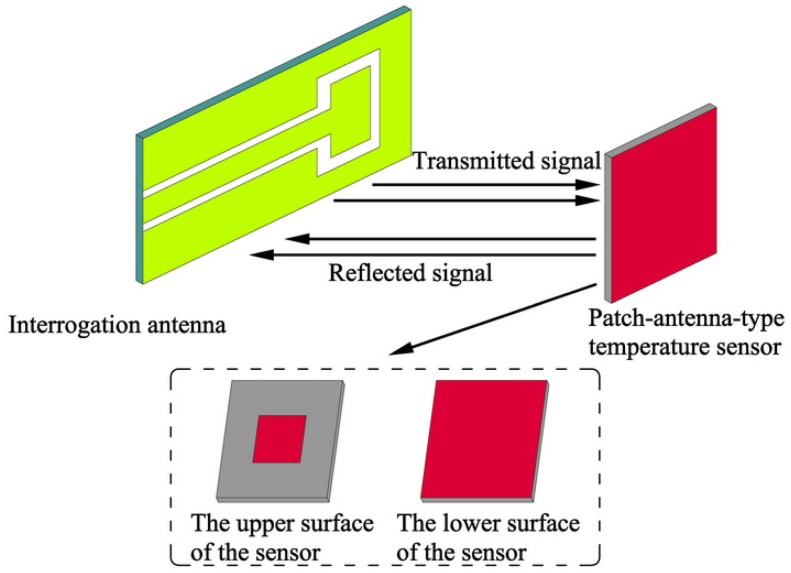
Schematic diagram of the patch antenna temperature sensor system.

**Figure 2:**
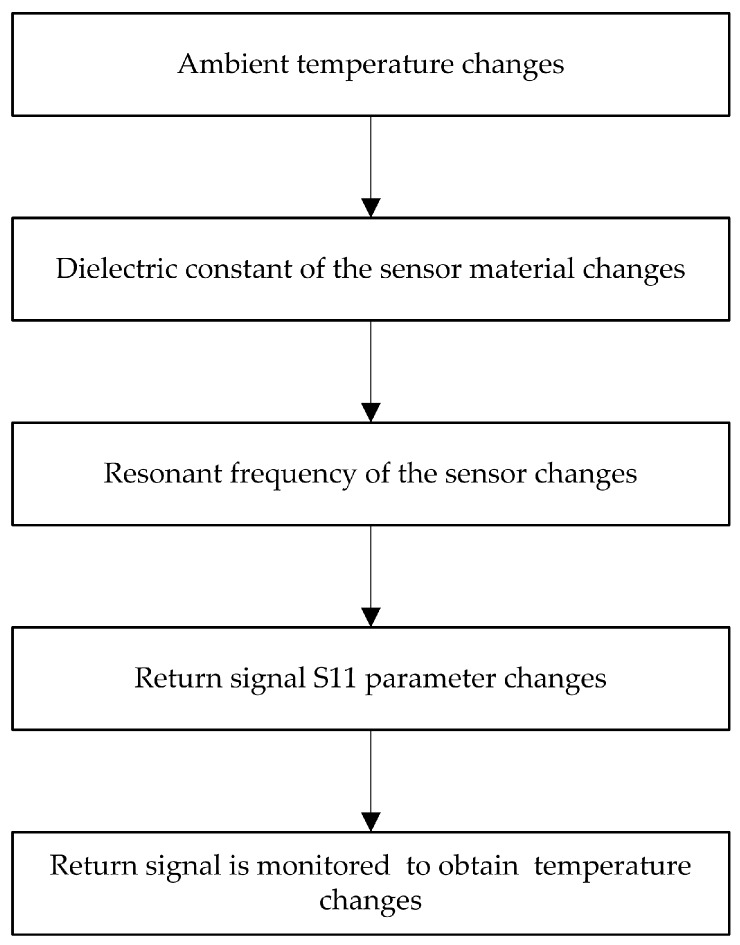
Patch antenna-type temperature sensor measurement process.

Figure 1 shows the schematic diagram of the patch antenna-type wireless passive temperature sensor system, which consists of a temperature sensor and an interrogation antenna. The temperature sensor is a resonant patch antenna consisting of an AlN ceramic substrate with a metal patch on its upper surface and a metal ground on its lower surface. An Al_2_O_3_-based patch loading slot antenna is used as the interrogation antenna, and a coplanar waveguide feed is adopted [43]. To sense the temperature, the antenna sends a sweep signal with a certain bandwidth to the temperature sensor; the frequency component is absorbed by the sensor when the frequency component of the swept signal is the same as the resonant frequency of the temperature sensor. The remaining part of the sweep signal will be reflected back and accepted by the interrogation antenna. This reflection from the temperature sensor can be displayed by a vector network analyzer (VNA), and it is displayed on the screen of the VNA as the return loss (*S*_11_) frequency curve; the maximum trough in this curve indicates the resonant frequency of the sensor. When the environmental temperature of the sensor changes, the dielectric constant of the sensor substrate will change, as the dielectric constant is related to the temperature. Furthermore, the resonant frequency of the patch sensor is a function of the dielectric constant, so it will change, and the resonant frequency signal detected by the interrogation antenna will reflect this change. Obviously, the resonant frequency of the sensor is a composite function of the ambient temperature, and thus the temperature can be extracted from the resonant frequency of the sensor. The measurement process of the temperature sensor is shown in Figure 2.

**Figure 3:**
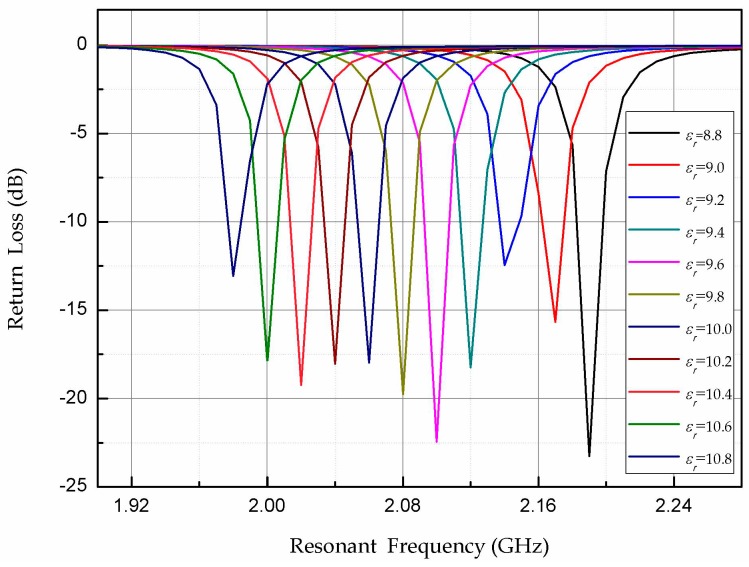
Simulated *S*_11_ curves of the sensor for different dielectric constants of the aluminum nitride (AlN) ceramic material.

**Figure 4:**
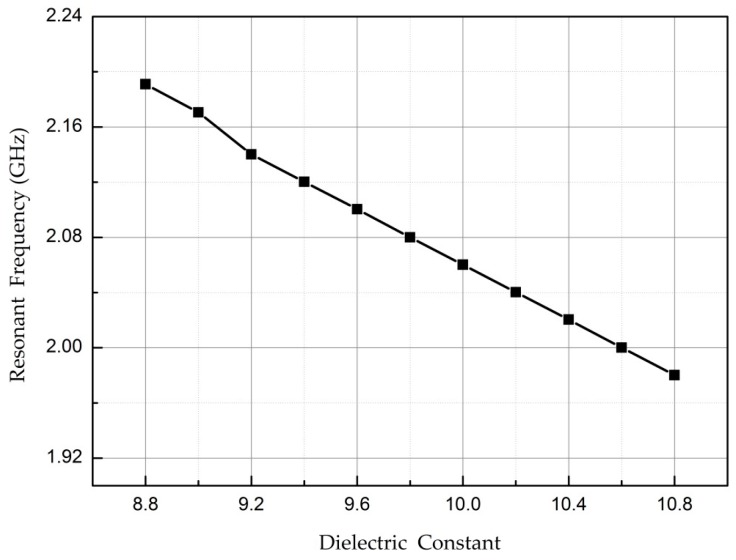
Simulated relationship between the dielectric constant and resonant frequency.

The high-frequency simulator structure (HFSS) simulation software is used to verify the temperature-sensing mechanism. In the simulation using HFSS, the dielectric constant of the AlN ceramic material is set to range from 8.8 to 10.8 (the dielectric constant of the AlN ceramic material increases gradually with increasing temperature, so the dielectric constant is gradually increased) [44]. Figure 3 shows *S*_11_ curves corresponding to different dielectric constants of the AlN substrate. The trough of the *S*_11_ curve corresponds to the resonant frequency of the sensor. Obviously, as the dielectric constant of the AlN substrate changes, the resonant frequency of the sensor changes. For a clearer view of the relationship between the resonant frequency of the temperature sensor and the dielectric constant of the material, the valley points in Figure 3 are extracted and plotted as a curve, as shown in Figure 4. The resonant frequency clearly decreases as the permittivity increases. Therefore, the principle of the sensor is verified.

## 3. Temperature Sensor Design

The patch antenna temperature sensor operates in ${\mathrm{TM}}_{01}$ mode, and the resonant frequency is calculated as follows:

(1) $$f_{r}=\frac{c}{2\left(L+2\Delta L\right)\sqrt{\varepsilon_{e}}}$$

where *c* is the speed of light in vacuum; L+2ΔL is the equivalent length of the radiation patch taking into account the edge effect [45]; and $\varepsilon_{e}$ is the effective permittivity of the substrate.

The width of the radiation patch is calculated as follows:

(2) $$W=\frac{c}{2f_{r}}{\left(\frac{\varepsilon_{r}+1}{2}\right)}^{-\frac{1}{2}}$$

where $f_{r}$ is the center frequency of the temperature sensor ($f_{r}=2.2\;\mathrm{GHz}$ at room temperature); and $\varepsilon_{r}$ is the dielectric constant of the AlN substrate ($\varepsilon_{r}=8.8$ at room temperature). Therefore, according to formula (2), *W* = 30.8 mm.

**Figure 5:**
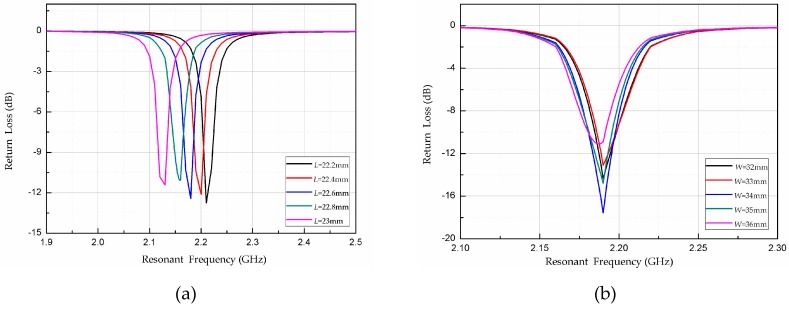
High-frequency simulator structure (HFSS) simulation results: **(a)** length *L*; and **(b)** width *W* of radiation patch.

The length of the radiation patch is calculated as follows:

(3) $$L=\frac{c}{2f\sqrt{\varepsilon_{e}}}-2\Delta L$$

where Δ*L* is the equivalent radiation gap length. Δ*L* and $\varepsilon_{e}$ are calculated as follows:

(4) $$\varepsilon_{e}=\frac{\varepsilon_{r}+1}{2}+\frac{\varepsilon_{r}-1}{2}{\left(1+12\frac{h}{w}\right)}^{-\frac{1}{2}}$$

(5) $$\Delta L=0.412h\frac{\left(\varepsilon_{e}+0.3\right)\left(\frac{w}{h}+0.264\right)}{\left(\varepsilon_{e}-0.258\right)\left(\frac{w}{h}+0.8\right)}$$

where *h* (= 1.0 mm) is the thickness of the AlN substrate. According to formulas (3), (4) and (5), the radiation patch length of the temperature sensor *L* is 22.9 mm. To improve the radiation efficiency of the sensor and reduce the reflection and transmission losses, the length *L* and width *W* of the sensor radiation patch were optimized on the basis of the theoretical value obtained using HFSS, as shown in Figure 5. The resonant frequency $f_{r}$ of the sensor decreases gradually with increasing length of the radiation patch; and the resonant frequency of the sensor is equal to the design center frequency of 2.2 GHz when *L* = 22.4 mm. The width of the radiation patch has little effect on the sensor; $S_{11}$ is smallest, and the radiation effect is largest, when *W* = 34 mm. The final dimensions of the optimized sensor are shown in Table 1.

## 4. Temperature Sensor Fabrication

**Figure 6:**
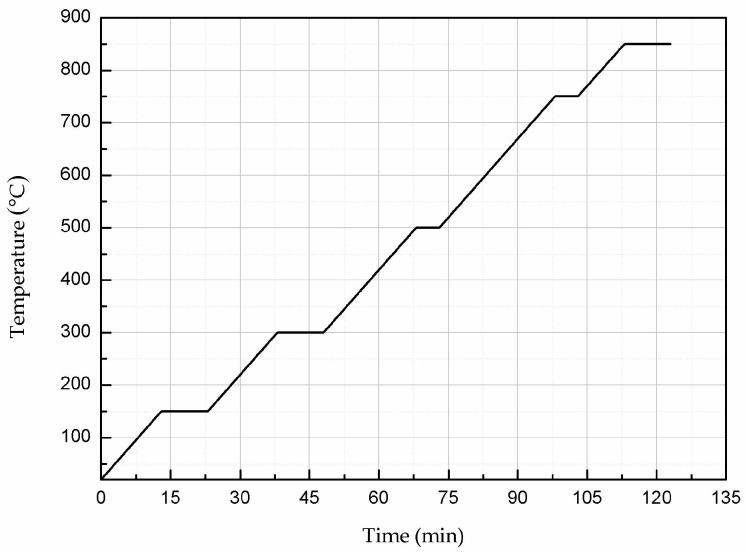
Sintering curve of the silver–palladium slurry.

**Figure 7:**
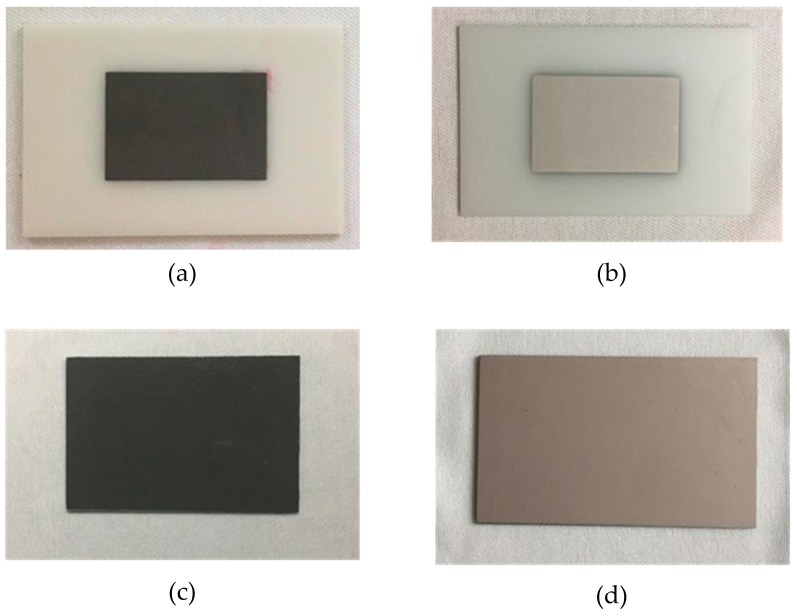
Patch antenna-type temperature sensor after each step of the fabrication process: (**a**) printed radiation patch; (**b**) sintered radiation patch; (**c**) printed metal layer; (**d**) sintered metal layer.

The substrate material of the sensor is AlN ceramic; the length, width, and thickness of the substrate are 44.8 mm, 68 mm and 1.0 mm, respectively. Silver–palladium metal paste was printed on the upper and lower surfaces of the AlN ceramic substrate by screen-printing technology to form the sensor’s radiation patch and metal layer (ground), respectively. In the silver–palladium paste used, the conductivity of palladium is less than silver. According to the formula of the skin depth ($\delta =\frac{1}{\sqrt{\pi f\sigma \mu_{1}}}$), the conductivity is inversely proportional to the depth of the skin. Therefore, when the conductivity of palladium is used, the skin depth is the largest and calculated to be 3.56 μm. The thickness of the radiation patch is 25 μm, much larger than the calculated value of the skin depth, so the effect of the skin effect can be neglected. After the printing was completed, the silver–palladium metal paste was sintered to solidify it and form a dense metal layer on the surface of the ceramic substrate. During the sintering process, the temperature was raised from room temperature (20 °C) to 850 °C at a rate of 10 °C/min for 123 min and then cooled naturally to room temperature. Figure 6 shows the sintering curve. The temperature sensor fabrication process is shown in Figure 7; the fabrication steps are as follows: (a) the radiation patch is plated on the surface of the cleaned AlN ceramic substrate; (b) the ceramic substrate with the radiation patch is sintered in a muffle furnace according to the sintering curve in Figure 6; (c) after the ceramic substrate is cooled to room temperature, the metal layer is printed on its lower surface; (d) the ceramic substrate with the metal layer is placed in the muffle furnace for sintering again, and after sintering is completed, it is cooled to room temperature.

## 5. Measurement and Discussion

**Figure 8:**
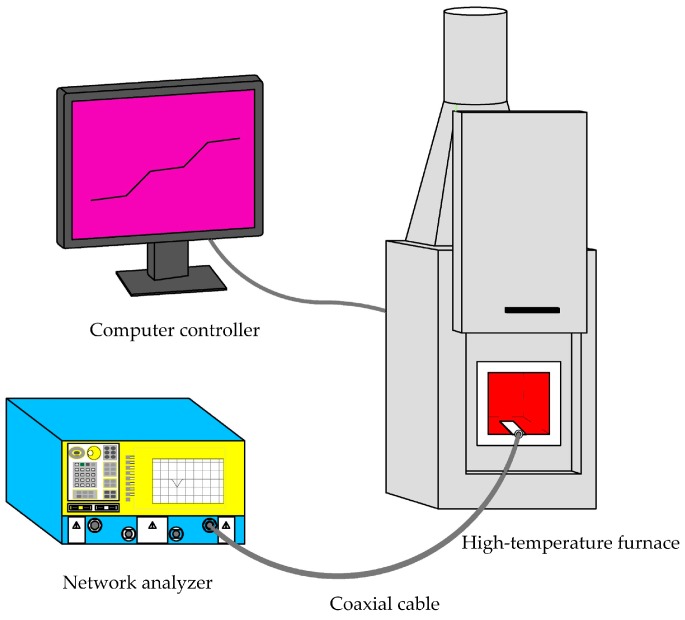
Illustration of the high-temperature testing system.

To test the performance of the prepared patch antenna temperature sensor, a high-temperature test system was built, as illustrated in Figure 8. The system consists of a computer controller, high-temperature heating furnace (Nabertherm, LHT08/16, Nabertherm GmbH, Lilienthal, Germany), and network analyzer (PNA Network Analyzer N5224A, 10 MHz–43.5 GHz, SolarWinds, Austin, TX, USA). The pre-set temperature curve is inputted to the software controlling the computer, and the software automatically sends the data to the furnace to control its temperature. The furnace is used to heat the internal interrogation antenna and temperature sensor. The interrogation antenna, which has a subminiature version A (SMA) adapter, is connected to the network analyzer through a coaxial line to monitor and display the return signal (*S*_11_).

**Figure 9:**
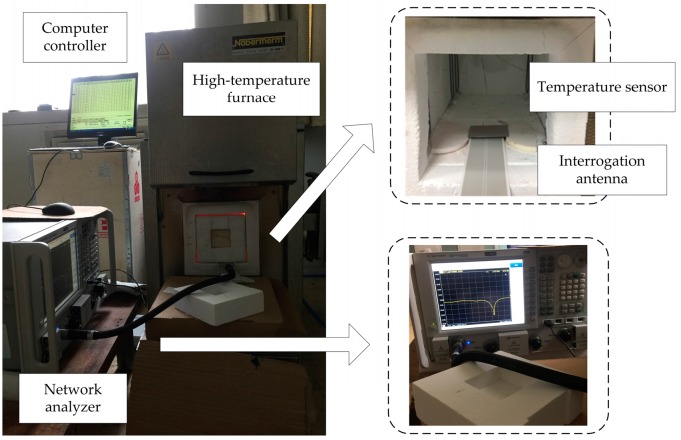
Temperature testing setup.

During the heating process, the temperature was increased at a rate of 10 °C/min; and when the temperature had changed by 100 °C and held for 10 minutes, the experimental data were recorded. In the testing system, high-temperature insulation material 50 mm thick was installed in the door of the furnace to improve the accuracy of the temperature and reduce the heat loss. Inside the furnace, the temperature sensor was placed parallel to the front of the interrogation antenna at a distance of 15 mm. The testing setup is shown in Figure 9.

**Figure 10:**
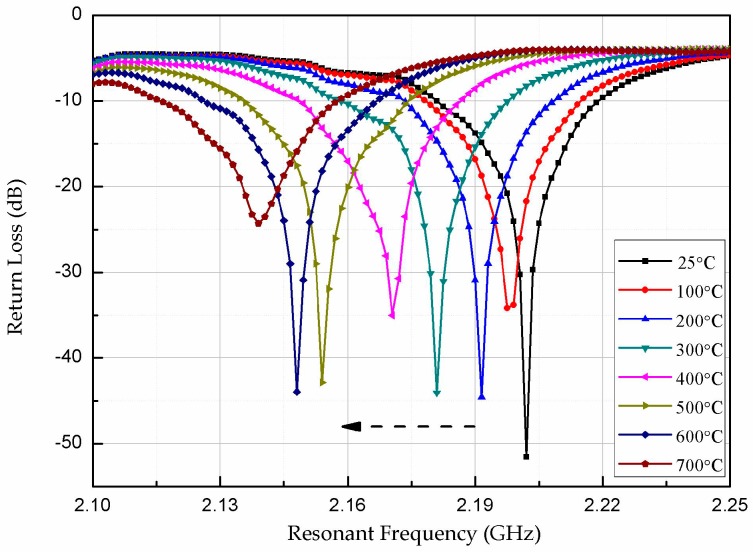
Return loss versus resonant frequency at various temperatures.

Figure 10 shows the resonant frequency curve of the patch antenna-type temperature sensor during heating. The valley of the curve gradually shifts to the left, and the resonant frequency of the sensor decreases gradually with increasing temperature. The resonant frequency of the temperature sensor is 2.20 GHz at room temperature, which is the same as the designed center frequency of 2.2 GHz, indicating agreement between the theoretical design and realized device.

**Figure 11:**
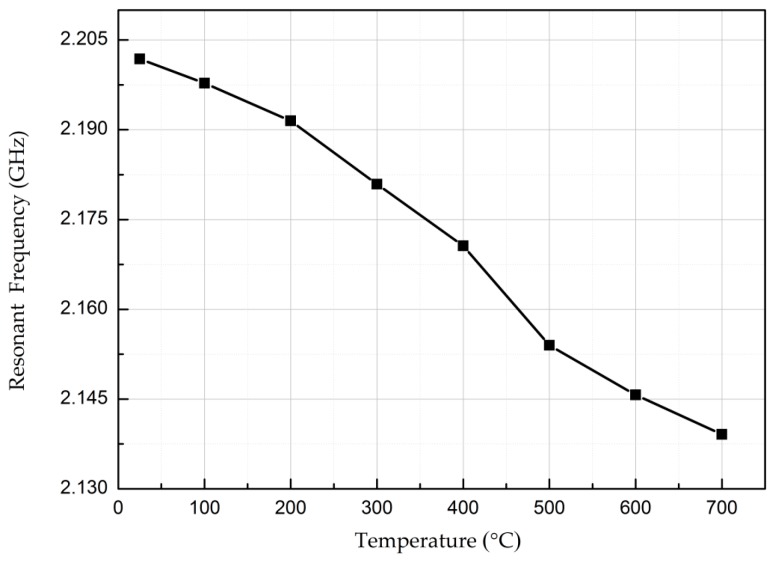
Valley resonant frequency versus temperature during heating.

The valley of the curve is extracted to clearly show the change in resonant frequency with increasing temperature, as shown in Figure 11. The resonant frequency of the temperature sensor changed from 2.20 GHz to 2.13 GHz as the temperature increased from room temperature (25 °C) to 700 °C. Therefore, the absolute sensitivity of the temperature sensor is $S_{f}=\frac{\Delta f}{\Delta t}=104.77\;\mathrm{KHz}/{}^{\circ}C$, and the resonant frequency varies by 3.2%. The thermal expansion coefficient of the AlN ceramic substrate is $4.6\times 10^{-6}/{}^{\circ}C$ for the temperature increase from 25 °C to 700 °C and reflects a size change of 0.3105% due to thermal expansion. Because the size change is significantly less than 3.2%, the change in the temperature sensor’s frequency is due mainly to the change in the dielectric constant.

**Figure 12:**
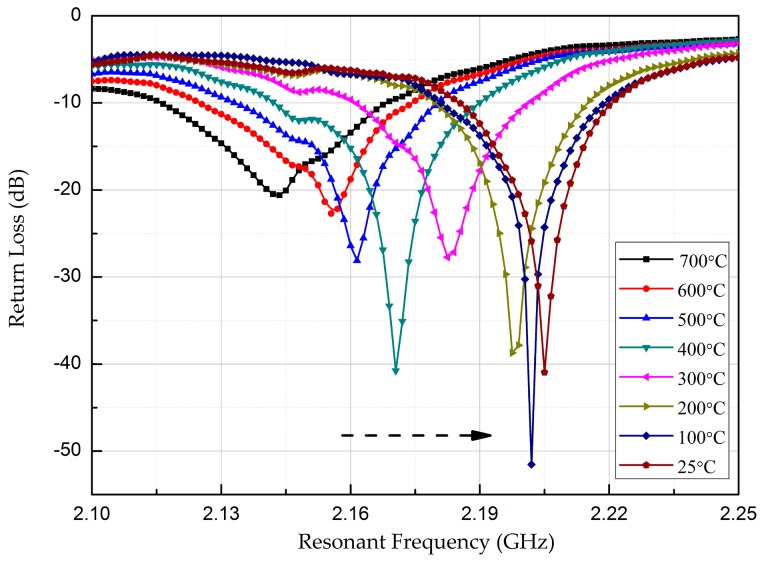
Return loss versus resonant frequency during cooling.

Figure 12 shows the relationship between the resonant frequency of the sensor and the temperature during cooling. The valley of the curve shifts gradually to the right, and the resonant frequency of the sensor increases gradually with decreasing temperature.

**Figure 13:**
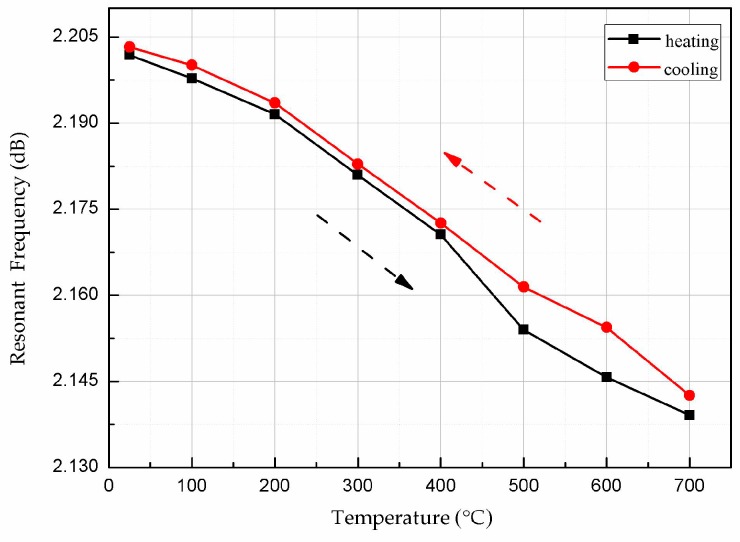
Valley resonant frequency versus temperature during heating and cooling.

The valley points of the curves during heating and cooling were extracted and plotted, as shown in Figure 13. The curves of the sensor in the heating and cooling processes are nearly the same, indicating that the output of the sensor at the same temperature deviates very little for measurement during heating and cooling. A slight deviation in the temperature profile at 500–600 °C is due to variations in the temperature control and ambient test environment. Therefore, the hysteresis error of the sensor is small at temperatures below 400 °C.

**Figure 14:**
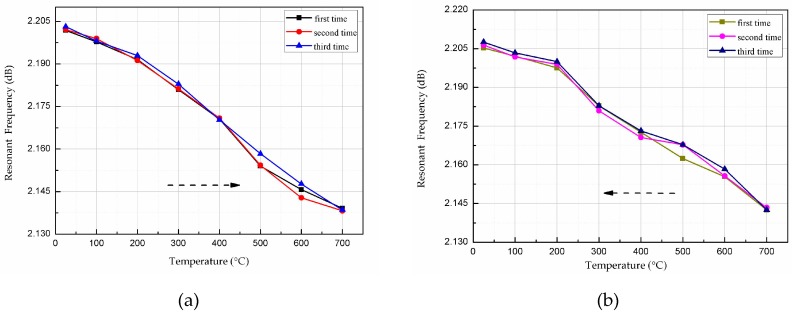
Sensor repeatability test curve: (**a**) heating process; (**b**) cooling process.

Using the same test system, the sensors were tested three times to verify the repeatability during heating and cooling. The valley points were extracted from the heating and cooling curves and are plotted in Figure 14a,b, respectively. Below 400 °C, the sensor exhibits very good repeatability, as the curves overlap almost completely. Therefore, the AlN-based patch antenna temperature sensor has excellent practical utility and test reliability below 400 °C.

## 6. Conclusions

This paper presents a patch antenna-type wireless passive temperature sensor made of an AlN ceramic material and silver–palladium metal paste. The sensor has advantages such as high-temperature operation, compactness, a simple structure, ease of processing, ease of integration, and low cost; furthermore, it can be applied in harsh high-temperature environments. The feasibility of applying an AlN material and a thick-film printing process to sensors for high-temperature measurement is proved by theoretical analysis, simulation, fabrication, and experiment. The resonant frequency of the sensor changes from 2.20 GHz to 2.13 GHz as the temperature is raised from room temperature (25 °C) to 700 °C, and the absolute sensitivity is 104.77 KHz/°C. Three measurements during heating and cooling were performed and showed that the sensor has good repeatability below 400 °C and a small hysteresis error. Our future work will improve the sensitivity and sensing distance of the sensor by improving the fabrication process and machining accuracy and designing a high-gain broadband interrogation antenna.

## Figures and Tables

**Table 1:** Patch antenna parameters.

Symbol	Parameter	Value (mm)
*L*	Patch length	22.4
*W*	Patch width	34
*H*	Substrate thickness	1.0
2*L*	Substrate length	44.8
2*W*	Substrate width	68
